
JOURNAL INFORMATION
==============================
NLM Title Abbreviation: Exp Physiol
Journal ID: Exp Physiol
NLM Journal ID: 9002940
Journal ID: EPH

Experimental Physiology

ISSN: 0958-0670
EISSN: 1469-445X
Publisher: Wiley

ARTICLE INFORMATION
==============================
PMCID: PMC12949104
DOI: 10.1113/eph.70238
Article ID: EPH70238
Article version: 1
Subjects: Issue Information, Issue Information

Issue Information

Electronic publication date: 2026 Feb 27
Publication date: 2026 Mar
Volume: 111
Issue: 3
First page: 1554
Last page: 1560
Copyright: © 2026 The Authors. Experimental Physiology published by John Wiley & Sons Ltd on behalf of The Physiological Society.
License: This is an open access article under the terms of the http://creativecommons.org/licenses/by/4.0/ License, which permits use, distribution and reproduction in any medium, provided the original work is properly cited.
License URL: https://creativecommons.org/licenses/by/4.0/

Figure count: 0
Table count: 0
Page count: 6

source-schema-version-number: 2.0
cover-date: 1 March 2026
details-of-publishers-convertor: Converter:WILEY_ML3GV2_TO_JATSPMC version:6.7.1 mode:remove_FC converted:28.02.2026

==============================

### Experimental Physiology

Open Access

Integration and Translation

Volume 111 | Issue 3 | 1 March 2026

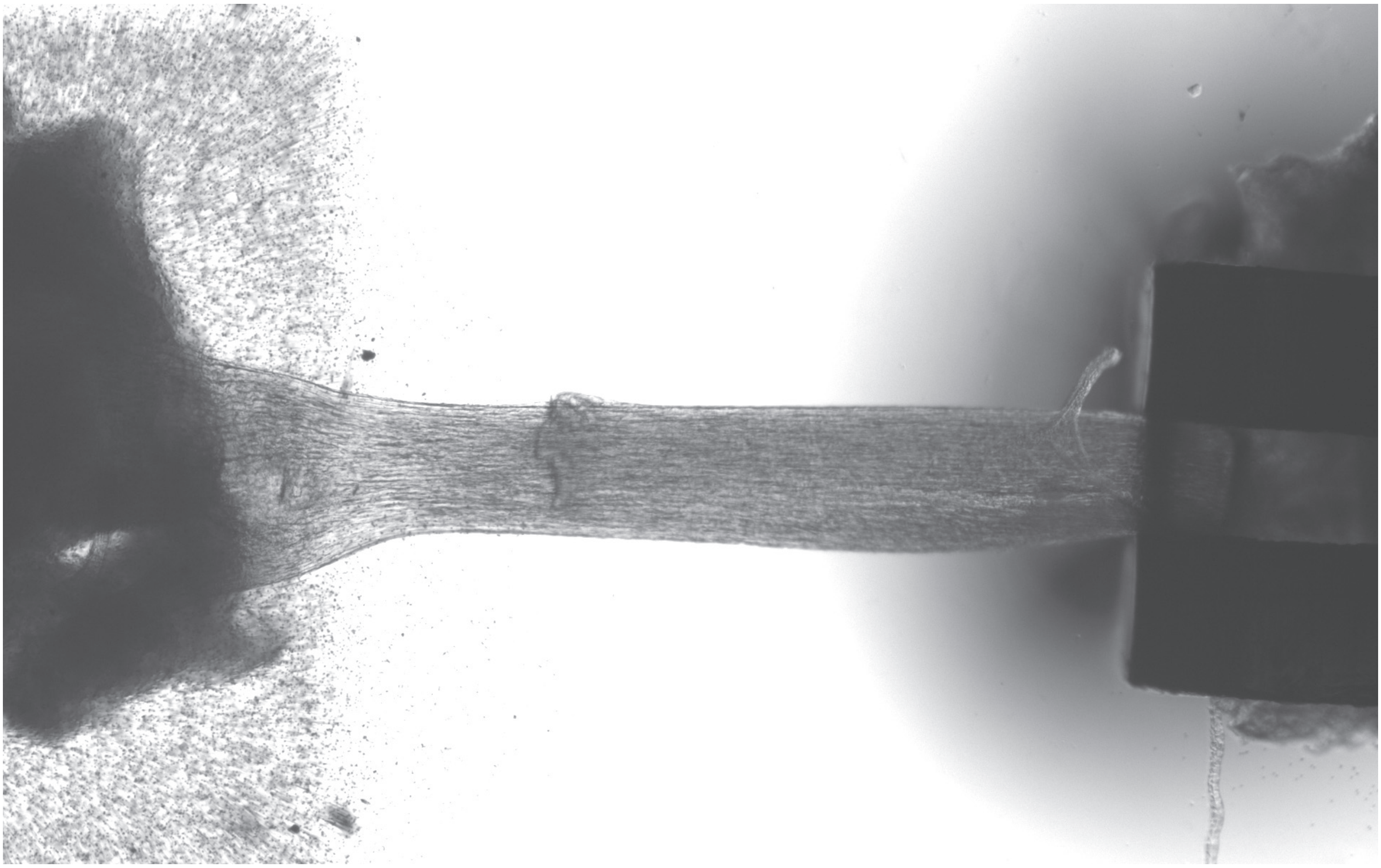

WILEY

The Physiological Society

150 YEARS 1876 2026 LEADING DISCOVERY SCIENCE

Experimental Physiology
Open Access
Integration and Translation

Volume 111/Issue 3/
1 March 2026

### EDITORIAL BOARD

**EDITOR-IN-CHIEF**
Damian Bailey *Pontypridd, UK*

**DEPUTY EDITOR-IN-CHIEF EUROPE**
Ronan Berg *Copenhagen, Denmark*

**DEPUTY EDITOR-IN-CHIEF USA**
David Poole *Manhattan, KS, USA*

**SENIOR ETHICS EDITOR**
Susan Barman *East Lansing, MI, USA*

**STATISTICAL EDITOR**
Cody Durrer *Copenhagen, Denmark*

**SENIOR REVIEWS EDITOR**
Toby Mündel *Ontario, Canada*

**CONNECTIONS EDITOR**
Prem Kumar *Birmingham, UK*

**SENIOR EDITORS**
Dana Almohazey *Dammam, Saudi Arabia*
Jill Barnes *Madison, WI, USA*
R. Matthew Brothers *Arlington, TX, USA*
Kanokwan 'Mandy' Bunsawat *Iowa City, IA, USA*
Kirstene Callø *Copenhagen, Denmark*
Katrina Colafella *Victoria, Australia*
Ellen Dawson *Liverpool, UK*
Colleen Deane *Southampton, UK*
Peter Zu Eulenburg *Munich, Germany*
Federico Formenti *London, UK*
Julien Ochala *Copenhagen, Denmark*
Shigehiko Ogoh *Kawagoe, Japan*
Jason Peart *Gold Coast, Australia*
Peter Rasmussen *Copenhagen, Denmark*
Lee Romer *London, UK*
Harry B. Rossiter *Los Angeles, CA, USA*
Bill Sheel *Vancouver, Canada*
Mike Stembridge *Cardiff, UK*

**REVIEWING EDITORS**
Tatsuro Amano *Niigata, Japan*
Claire Badenhorst *Palmerston North, NZ*
Tracy Baynard *Boston, MA, USA*
Hannah Caldwell *Copenhagen, Denmark*
Havovi Chichger *Cambridge, UK*
Silvia Conde *Lisbon, Portugal*
Joseph Costello *Portsmouth, UK*
Jennifer Davies *Cardiff, UK*
Judy Delp *Tallahassee, FL, USA*
Aimee Drane *Cardiff, UK*
Esther Dupont-Versteegden *Lexington, KY, USA*
Andrea Fellet *Buenos Aires, Argentina*
Carrie Ferguson *Torrance, CA, USA*
Igor Fernandes *Niteroi, Brazil*
Ryan Hoiland *Kelowna, Canada*
Seth Holwerda *Kansas City, KS, USA*
Anna Hudson *Adelaide, Australia*
Matthew Kay *Washington, DC, USA*
James Lang *Ames, Iowa, USA*
Jacqueline Limberg *Columbia, MO, USA*
Fiona McBryde *Auckland, NZ*
Carmen De Miguel *Birmingham, AL, USA*
Niels Olesen *Copenhagen, Denmark*
Leah Reznikov *Gainesville, FL, USA*
Kleiton Silva *Camden, NJ, USA*
Sarah Short *Iowa City, IA, USA*
Nathaniel Szewczyk *Nottingham, UK*
Can Ozan Tan *Boston, MA, USA*
Mike Tymko *Vancouver, Canada*
Kai Yang *Guangzhou, China*
Song Yao *Melbourne, Australia*
Gensheng Zhang *Hangzhou, China*

**FUTURE LEADERS**
Edward Ashworth *Basel, Switzerland*
Sophie Broome *Melbourne, Australia*
Callum Brownstein *Newcastle, UK*
Daniel Craighead *Minneapolis, MN, USA*
Robert Lakin *Toronto, Canada*
Jedd Pratt *Manchester, UK*
Fabio Sarto *Padua, Italy*
Hollie Speer *Canberra, Australia*

**CONSULTANT EDITORS**
Richard Burden *Bisham, UK*
Nic Green *Bedfordshire, UK*
Julie Greeves *Salisbury, UK*
Denis Noble *Oxford, UK*
Nanduri Prabhakar *Chicago, IL, USA*

**MANAGING EDITOR**
Alex Stewart (astewart@physoc.org)

**PEER REVIEW COORDINATOR**
Diana Jones (ephjournal@physoc.org)

**EDITORIAL OFFICE**
The Physiological Society
Hodgkin Huxley House
30 Farringdon Lane, Clerkenwell
London EC1R 3AW

**PRODUCTION OFFICE**
K. Nandini (EPH@wiley.com)

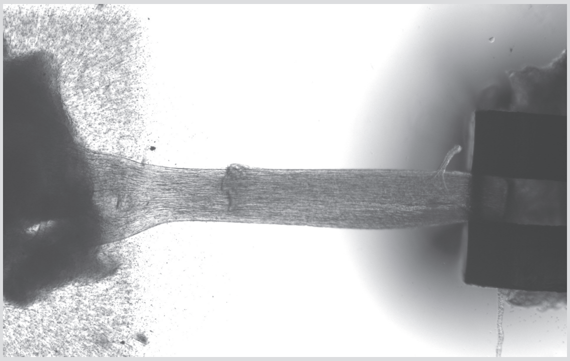

**Cover Illustration**
Cardiac muscle sample being mechanically tested in a new high-throughput instrument. Provided by Murphy et al. Pages 632–642.

**Open Access and Copyright**
All articles published by Experimental Physiology [submitted after 18 October 2022] are fully open access: immediately freely available to read, download, and share. All articles [accepted from 18 October 2022] are published under the terms of a Creative Commons license.
Copyright on any research article published by Experimental Physiology is retained by the author(s).
Further information about open access licenses and copyright can be found at: https://authorservices.wiley.com/author-resources/Journal-Authors/licensing/licensing-info-faqs.html#4. Information on the licenses used by Experimental Physiology can be found at: https://physoc.onlinelibrary.wiley.com/journal/1469445X.

For submission instructions and all other information visit: https://physoc.onlinelibrary.wiley.com/journal/1469445X

The Physiological Society and The Physiological Society logo are trade marks belonging to The Physiological Society and are registered in the UK and in the EU Community, respectively.

*Experimental Physiology* publishes high quality, original, physiological research papers that give novel insights into fundamental homeostatic and adaptive responses in health and papers that further our knowledge of pathophysiological mechanisms in diseases.

Experimental Physiology
Open Access
Integration and Translation

### INFORMATION AND GUIDANCE FOR AUTHORS

**Full version online at http://ep.msubmit.net/cgi-bin/main.plex?form_type=display_requirements**

### ORIGIN

*The Quarterly Journal of Experimental Physiology*, founded in 1908 by Sir Edward Sharpey-Schafer, Professor of Physiology, University of Edinburgh, became a publication of The Physiological Society in 1981. The title was changed to *Experimental Physiology* in 1990.

### SCOPE

*Experimental Physiology* publishes research papers that report novel insights into homeostatic and adaptive responses in health, as well as those that further our understanding of pathophysiological mechanisms in disease. We encourage papers that embrace the journal's orientation of translation and integration, including studies of the adaptive responses to exercise, acute and chronic environmental stressors, growth and aging, and diseases where integrative homeostatic mechanisms play a key role in the response to and evolution of the disease process. Examples of such diseases include hypertension, heart failure, hypoxic lung disease, endocrine and neurological disorders. We are also keen to publish research that has a translational aspect or clinical application. Comparative physiology work that can be applied to aid the understanding human physiology is also encouraged.

Manuscripts that report the use of bioinformatic, genomic, molecular, proteomic and cellular techniques to provide novel insights into integrative physiological and pathophysiological mechanisms are welcomed.

We are also interested in methodological papers reporting important new developments in physiological techniques that could lead to significant new insights.

Papers which observe a significant new physiological behaviour or phenomenon which has the potential to alter the understanding of that field, but which have not yet identified the mechanisms may also be considered. Such papers should demonstrate the experimental examination and exclusion of a number of plausible mechanisms for the important novel phenomenon.

### Article Types

In addition to original research, *Experimental Physiology* consider the following article types for publication:

*Case studies, Short Communication, Connections, Reviews, Full Reviews, Hot Topic Reviews Myths and Methodologies and Symposium Reports.*

Review articles are normally by invitation but proposals may be submitted for consideration.

Commentary articles in the form of Viewpoints and Letters are also published.

For full guidelines on all published article see full guidelines.

C O P E COMMITTEE ON PUBLICATION ETHICS

This journal is a member of and subscribes to the principles of the Committee on Publication Ethics

### ETHICS OF EXPERIMENTATION

**Animal Experiments** - see full journal policy at https://physoc.onlinelibrary.wiley.com/hub/animal-experiments

Experimental Physiology endorses the ARRIVE Guidelines for reporting *in vivo* experiments: http://onlinelibrary.wiley.com/doi/10.1113/expphysiol.2010.053793/full

Authors of papers submitted to Experimental Physiology must adhere to the journals' principles and standards for reporting animal experiments.

Authors should read these guidelines to ensure that their research and experiments adhere to our exacting standards.

Euthanasia/humane killing: Animals must be killed using methods approved for that species, stage of development and size. The standards required by the journals are those of the UK/European Union legislation, set out in in ASPA Schedule 1 in the UK and in Annex IV in the European Directive 2010/63/EU. Methods not on Schedule 1 or Annex IV could be permissible but even where the method used conforms with national guidelines, the onus is on the author to provide scientific evidence to demonstrate that it is at least as humane as those on Schedule 1 and Annex IV.

Our guidelines have been outlined in the Editorial 'Principles and standards for reporting animal experiments in Experimental Physiology'.

**Confirmation of compliance**

Authors must state in the Methods section that the investigators understand the ethical principles under which the journal operates and that their work complies with the animal ethics checklist as outlined in the Editorial.

**Human Experiments** - See full journal policy at https://physoc.onlinelibrary.wiley.com/hub/human-experiments

The Editorial Boards are concerned that all work published in The Journal of Physiology and Experimental Physiology should have been conducted according to the highest standards of safety and ethics. Legislation and accepted practice concerning human experimentation vary from country to country and consequently it is difficult to define absolute requirements. However, work with human subjects should conform to the standards set by the Declaration of Helsinki (last modified in 2013), the Medical Research Council's online guidance on the use of human tissue, and the guidelines set out below. The objectives must be to ensure that all risks are minimized and that subjects are not injured and do not feel they have been abused as a result of participating in the study. Any definition of abuse will include excessive or unexpected pain or discomfort experienced during the experiment. Note, in the case of experiments involving minors, any risks must have been considered absent or minimal, and evidence must be presented that the experiments were performed with the understanding and consent of the legal guardian.

All manuscripts must contain statements indicating that (i) informed consent has been obtained, preferably in writing, (ii) studies conformed to the standards set by the Declaration of Helsinki or the version that was in place at the time of the experiments, NOTE: If the research study was registered (clause 35 of the Declaration of Helsinki) the registration database should be indicated, else the lack of registration should be noted as an exception (e.g. The study conformed to the standards set by the Declaration of Helsinki, except for registration in a database.); and (iii) the procedures have been approved by the local ethics committee.

### DISCLAIMER

The Publisher, the Physiological Society and Editors cannot be held responsible for any errors in or any consequences arising from the use of information contained in this journal. The views and opinions expressed do not necessarily reflect those of the Publisher, the Physiological Society or Editors, neither does the publication of advertisements constitute any endorsement by the Publisher, the Physiological Society, Editors, or Authors of the products advertised.

Wiley Open Access articles posted to repositories or websites are without warranty from Wiley of any kind, either express or implied, including, but not limited to, warranties of merchantability, fitness for a particular purpose, or non-infringement. To the fullest extent permitted by law Wiley disclaims all liability for any loss or damage arising out of, or in connection with the use of or inability to use the content.

Experimental Physiology

Vol 111.3

### Contents of volume 111.3

1 March 2026

### EDITORIAL

587 Unlimited adaptations of muscle fibres to exercise; are you kidding me?
*Hans Degens and Paul Hendrickse*

590 The art of sharing: From research to outreach in the social media era
*Milan Mohammad, Abdullahi A. Mohamed, Jari Dahmen, Jonathan J. Bjerre-Bastos and Andreas Pihl*

### VIEWPOINT

596 Sildenafil helps the beat go on
*Jules C. Hancox*

598 Induction of ischaemic cardiomyopathy in mice without off-target effects
*Mark T. Waddingham and James T. Pearson*

### SHORT COMMUNICATION

600 Evidence of racial differences in peripheral blood pressure, central haemodynamics and arterial stiffness between young Black and White women
*Michele N. D'Agata, Elissa K. Hoopes, Alexs A. Matias, Mackenzie L. Rattigan, Freda Patterson and Melissa A. Witman*

608 A new method for non-invasive determination of effective pulmonary blood flow and cardiac output in spontaneously breathing subjects
*Andras Gedeon, Jakob Jansson, David Patrickson and Mats Wallin*

614 Continuous carbon dioxide monitoring in the exhaled breath of mechanically ventilated rats
*Christin Wenzel, Silke Borgmann, Bernd Flamm, Lea Kuhn, Stefan Schumann, Johannes Schmidt, Johannes Spaeth and Sashko Spassov*

620 A new method for bedside determination of effective lung volume and functional residual capacity
*Andras Gedeon, Jakob Jansson, David Patrickson and Mats Wallin*

### CASE REPORT

625 Suppression of premature ventricular complexes with the PDE5 inhibitor sildenafil: First clinical experience
*David C. Hutchings, Christopher P. Denton, Luigi Venetucci and Andrew W. Trafford*

### METHODS AND TECHNIQUES

632 Measurement and control of mechanics of cardiac trabeculae secured by light-curable hydrogel
*Emily J. Clark Murphy, Toan Pham, June-Chiew Han, Kenneth Tran, Khoon S. Lim, Poul M. F. Nielsen and Andrew J. Taberner*

### REVIEW ARTICLE

643 Sweat evaporation in humans: A molecular and thermodynamic perspective
*Edward T. Ashworth*

653 Ouabain-induced hypertension in rats: Mechanisms, variability and translational implications
*Priscilla Rodrigues O. Feijó and Luis Eduardo M. Quintas*

### RESEARCH ARTICLE

### CARDIOVASCULAR CONTROL

674 Ageing attenuates regional vasoconstriction during acute lowering of upper and lower limbs
*John D. Akins, Jeung-Ki Yoo, Dan-Dan Sun, Rosemary S. Parker, Marcus A. Urey, Steven A. Romero, Justin S. Lawley, Satyam Sarma, Wanpen Vongpatanasin, Craig G. Crandall and Qi Fu*

684 Systolic-dominant coronary flow in rats and mice: Challenging the diastolic paradigm across conscious and anaesthetized states
*Heidi L. Lujan, Theodore W. Kurtz and Stephen E. DiCarlo*

700 ZFP36 inhibits the pro-apoptotic effects of transforming growth factor $\beta$1 on mitral valve interstitial cells via the GTP-binding protein 6 pathway in mitral valve prolapse
*Meng Zhao, Zhaoyi Zhu, Yawei Dai, Li Jiang, Qihan Wen, Yingjie Zhang, Qingyang Shi, Yihu Tang, Jingxin Zhou and Yanhu Wu*

714 Cardioprotection via vagus nerve stimulation preconditioning: Reducing ischaemia–reperfusion injury and arrhythmic risk
*Feng Hu, Yali Wang, Guangyu Li, Guangyu Wang, Qi Zhuang, Jinyao Jiang, Danfeng Hu, Lihui Zheng, Yan Yao, Minhua Zang and Jun Pu*

727 Exploring physiological factors underlying individual differences in exercise-induced blood pressure responses
*Shigehiko Ogoh, Ryosuke Takeda, Narumi Kunimatsu, Hayato Tsukamoto, Ai Shimada, Tomoki Watada, Marina Feeley, Taichi Nishikawa, Marino Karaki, Kohei Watanabe and Tadayoshi Miyamoto*

738 Optimizing the predictive validity of NIRS kinetic profiles to predict aerobic capacity from a resting skeletal muscle occlusion test
*Heru Syarli Lesmana, Kyohei Marume and Justin S. Lawley*

### ENDOCRINOLOGY AND METABOLISM

751 Impact of combined ginsenoside-MC1 and irisin on mitochondrial apoptosis in diabetic rats with hepatic reperfusion injury: Role of AMPK/JNK signalling
*Jie Lin, Lei Han, Zhigang Ma, Bo Yuan and Yabin Yu*

765 Modelling the effect of varying metabolic rate and cardiac output on estimated tissue and blood $O_2$ and $CO_2$ levels in an extreme deep-diver, the goose-beaked whale (*Ziphius cavirostris*)
*Andreas Fahlman, Gregory S. Schorr, David A. Sweeney, Brenda K. Rone, Shannon N. Coates, Austin S. Allen, Lucia M. Martín López, Susan M. Jarvis and Erin A. Falcone*

### ENVIRONMENTAL AND EXERCISE

786 Validation of muscle oxygenation kinetics to predict aerobic fitness and exercise transition thresholds
*Heru Syarli Lesmana, Ben Schroeder, Kyohei Marume, Patrick Rodrigues and Justin S. Lawley*

798 Trimethylamine *N*-oxide is elevated in postmenopausal women relative to age-matched men and premenopausal women among individuals with obesity
*Daniel J. Battillo and Steven K. Malin*
809 Imposed expiratory resistance, dynamic hyperinflation and locomotor power and fatigue
*Jonathan Cunha, Antoinette Domingo, Fred W. Kolkhorst, Harry B. Rossiter and Daniel T. Cannon*
820 Haematological adaptations to high-altitude and heat acclimation training in elite male cyclists
*Claes Cubel, Magnus B. Klaris, Joakim V. Larsen, Raphaël Faiss, Lars Nybo and Carsten Lundby*
834 Commuting by bicycle (vs. by car) is associated with improved aerobic power, microvascular function and diminished $CO_2$ output in the atmosphere
*Caterina Ursella, Giovanni Baldassarre, Lucrezia Zuccarelli, Federica Caponnetto, Francesco Curcio, Mattia D'Alleva, Maria De Martino, Elisabetta Fontanini, Andrea Palomba, Antonio Paolo Beltrami, Federico Formenti and Bruno Grassi*
849 Human adaptation to high-altitude: A contemporary comparison of the oxygen cascade in Andean, Tibetan and Ethiopian highlanders
*Ayechew A. Getu, Melissa Ilardo, Joshua C. Tremblay, Jay M. J. R. Carr, Vitalie Faoro and Philip N. Ainslie*
866 Exercise-induced effects on atherogenesis and tryptophan catabolism via the kynurenine pathway in an HIV-associated atherosclerosis mouse model
*Marcus V. S. Rangel, Mohammad Afaque Alam, Mohammad Islamuddin, Zheng Chen, Xuebin Qin and Juliana P. Borges*
877 Maximal strength and voluntary activation of adductor pollicis after a single session of acute intermittent hypercapnia or acute intermittent hypoxia
*Anandit J. Mathew, Harrison T. Finn, Chiettha Prajnadewie, Simon C. Gandevia, Janet L. Taylor and Jane E. Butler*
893 Optimising exercise intensity for gut health: Effect on microbiota composition, barrier integrity and inflammation in male Wistar rats
*Nova Sylviana, Putri Karisa, Nur Faizah Romadona and Imam Megantara*
904 Acute metabolic responses to high-intensity interval training in men with overweight or obesity: Does the exercise modality matter?
*Annaëlle Couvert, Mélanie Rance, Eric Doré, Vincent Martin, Duane Beraud, Claire Morel, Bruno Pereira, Antonio Herbert Lancha Junior and Nathalie Boisseau*
916 Authentic assessment meets sustainable development: Bringing meaning to undergraduate Physiology education
*Mary McGahon, Sarah Geraghty, Clare Foy and Sean Roe*

### GI AND EPITHELIAL

930 The effects of short-term consumption of a Western diet on aerobic exercise performance in bank voles with inherently distinct metabolic rates
*Alaa Hseiky, Edyta T. Sadowska and Paweł Koteja*

### HEART/CARDIAC MUSCLE

946 Altered peripheral *CRY1* gene expression may contribute to both organic and functional gastrointestinal disease
*Sophie Fowler, Gemma M. Paech, Emily C. Hoedt, Jennifer C. Pryor, Cheenie Nieva, Jessica K. Bruce, Prema M. Nair, Guy D. Eslick, Simonne Sherwin, Peter Pockney, Nicholas J. Talley, Grace L. Burns and Simon Keely*
963 Atrial fibrillation, sinoatrial and atrioventricular node dysfunction in a mouse model of heart failure with preserved ejection fraction
*Bernadin Ndongson-Dongmo, Reinhard Bauer and Finn Olav Levy*
978 Left ventricular morphological and functional predictors of $\dot{V}_{O_2 peak}$: A 3-year observational study
*Viswanath B. Unnithan, Alexander Beaumont, Thomas Rowland, Keith George, Antonio Dello Iacono, Nicholas Sculthorpe, Rachel N. Lord and David L. Oxborough*
988 Antenatal betamethasone impairs markers of cardiac development and function in near-term lambs
*Reza Amanollahi, Ashley S. Meakin, Stacey L. Holman, Vicki L. Clifton, Kent L. Thornburg, Michael D. Wiese, Kathryn L. Gatford, Mitchell C. Lock and Janna L. Morrison*
1008 Computational study of the excitation of human induced pluripotent stem cell-derived cardiomyocytes
*Roshni Shetty, Raffi Samurkashian and Leslie Tung*
1039 Establishment of a mouse model for ischaemic heart failure induced by coronary microembolization via left ventricular oil injection
*Lang Pei, Yusheng Hong, Qiuyue Wu, Jianyi Lv, Changlong Li, Xueyun Huo, Xin Liu, Di Zhang, Meng Guo, Zhenwen Chen and Xiaoyan Du*
1058 Distinct profiles of mitochondrial bioenergetics and redox balance in left atrial and ventricular myocardium in the healthy rat heart
*Tingting Fang, Alex Chan, Janice Chew-Harris and Toan Pham*

### MUSCLE

1069 EuroSCORE II: Current limitations and physiological gaps in risk stratification
*Jing Yong Ng, Eu Fon Tan, Marsioleda Kemberi, Eduardo Urgesi, Matti Jubouri, Damian M. Bailey, Mohamad Bashir and Wael I. Awad*
1081 Mapping the variability of *in vivo* rat uterine electrical properties throughout the oestrous cycle
*Mathias W. Roesler, Amy S. Garrett, Leo K. Cheng and Alys R. Clark*
1093 Downregulation of microRNA-494 drives mitochondrial biogenesis and function in trained muscle
*Natália Pálešová, Klára Gabrišová, Jana Babulicová, Patrik Krumpolec, Zuzana Kovaničová, Tímea Kurdiová, Salvatore Modica, Christian Wolfrum, Jozef Ukropec, Barbara Ukropcová and Miroslav Baláž*
1109 The impact of the time of day on muscle and metabolic responses to resistance exercise in healthy adults: A randomised controlled trial
*Anas Dighriri, Hannah Lithgow, Brendan Gabriel, Mazin Altuwrqi, Emma Dunning, Lynsey Johnston, James G. Boyle, Greig Logan and Stuart R. Gray*
1120 Cardiac-specific Kv1.1 deficiency alters cardiomyocyte electrophysiology without modifying overall cardiac function or arrhythmia susceptibility
*Kelsey Halvorson, Man Si, Krystle Trosclair, Ellen Aughenbaugh, Maxine Parkinson, Nicole M. Gautier-Hall, Megan Watts, Frederica Kizek, Md. Shenuarin Bhuiyan, Paari Dominic, Kathryn A. Hamilton and Edward Glasscock*
1134 Impaired nitric oxide-dependent endothelial function in young male individuals with obesity before the onset of symptoms and complications
*Lucrezia Zuccarelli, Giovanni Baldassarre, Adele Bondesan, Diana Caroli, Roberta De Micheli, Gabriella Tringali, Thomas Favaretto, Joanna Suraj-Prazmowska, Anna Kurpinska, Joanna Majerczak, Stefan Chlopicki, Jerzy A. Zoladz, Alessandro Sartorio and Bruno Grassi*

1150 Steepest near-infrared spectroscopy-derived deoxygenation slopes during arterial occlusions provide more reliable assessments of muscle mitochondrial capacity
*Guillaume Costalat, Benoît Sautillet, Grégoire P. Millet, Clément Unal, Abd-Elbasset Abaïdia, Abdellah Hassar and Maryne Cozette*
1165 The effect of artificial gravity on the outcome of a two-week resistance vibration exercise programme: BRAVE study
*Igor B. Mekjavic, Riccardo G. Sorrentino, Jack Fortune, Jason T. Fisher, Lydia Tsoutsoubi, Leonidas G. Ioannou, Andrej Vovk, Matej Supej, Adam C. McDonnell and Urša Ciuha*
1181 Limited musculoskeletal benefits of artificial gravity combined with cycling during bed rest: Results from the BRACE study
*Mirko Mandić, Tommy R. Lundberg, Jean-Pol Frippiat, Adam C. McDonnell, Igor B. Mekjavić, Marie-Pierre Bareille, Rebecca Billette de Villemeur and Rodrigo Fernandez-Gonzalo*

### NEUROSCIENCE

1191 Use of transcutaneous spinal cord stimulation to explore inhibitory and facilitatory circuits in muscles of the human lower limb
*Julia Sordet, Maria Papaiordanidou, Nicolas Amiez, Ioannis Amiridis, Jean-Pierre Quenot and Alain Martin*
1210 Stroke and motor outcomes are associated with regional and age-specific changes in periodic and aperiodic cortical activity
*Asher J. Albertson, Eric C. Landsness, Margaret Eisfelder, Brittany M. Young, Bradley Judge, Matthew R. Brier, Matthew J. Euler, Steven C. Cramer, Jin-Moo Lee and Keith R. Lohse*
1228 Evaluation of metabolism-related molecules in rat model of autism spectrum disorders
*Süeda Tunçak, Ayşen Çakır, Bülent Gören and Nevzat Kahveci*
1242 Frequency responses of human magnetophosphene perception thresholds during dark adaptation point to rod modulation
*Nicolas Bouisset, Andres Carvallo, Sebastien Villard, Ilkka Laakso and Alexandre Legros*
1253 Spontaneous $Ca^{2+}$ signals in the developing mammalian cochlea of live mice under different anaesthetic regimes
*Francesca De Faveri, Walter Marcotti and Federico Ceriani*
1268 Temporal summation in human peripheral axons when stimulated transcutaneously with a 10-kHz waveform
*Billy L. Luu, Harrison T. Finn, Terry Trinh, Simon C. Gandevia, Martin E. Héroux and Jane E. Butler*
1279 Changes in corticospinal excitability in response to mediolateral gait instability
*Raven O. Huiberts, Sjoerd M. Bruijn and Jennifer L. Davies*
1299 Correlated amplitudes of potentials evoked in homologous muscles by magnetic stimulation reveal positive covariation of corticospinal output
*Richard G. Carson*
1313 Serendipitous compound action potential oscillations reveal glycolytic astrocyte and oxidative axon interstitial $K^+$ buffering in central white matter
*Amy J. Hopper and Angus M. Brown*
1330 Impact of long-term maternal high-fat, high-sugar feeding on gastric vagal afferent responses and feeding behaviour in mouse offspring
*Georgia S. Clarke, Hui Li, Elaheh Heshmati, Kelly M. Gembus, Stephanie E. O'Hara, Lisa M. Nicholas, Kathryn L. Gatford and Amanda J. Page*

### RENAL

1340 *Fmr1* knockout disrupts multiple intrinsic properties via reduced HCN channel activity in mediodorsal thalamocortical neurons
*Gregory J. Ordemann, Polina Lyuboslavsky, Alena Kizimenko and Audrey C. Brumback*
1365 Impact of diet and exercise in growth restricted female rats on nephron endowment in male fetuses
*Jessica F. Briffa, Sogand Gravina, Viktoria F. Richter, Dayana Mahizir, Kristina Anevska, Yeukai T. M. Mangwiro, James S. M. Cuffe, Fadi Charchar, Glenn D. Wadley, Deanne H. Hryciw, Karen M. Moritz and Mary E. Wlodek*

### RESPIRATORY

1387 Early acclimatization to high altitude: Acid–base and fluid balance dynamics during the first 2 days at 3100 m
*Elisabeth Skalla, Benedikt Treml, Sasa Rajsic, Michael Schreinlechner, Zoran Bukumirić, Klaus Berek, Alexander Egger, Johann Knotzer, Martin Burtscher and Axel Kleinsasser*
1397 Sex differences in loop gain measured via superimposed end-expiratory breath holds and inspired steady-state hypoxia
*Benjamin W. L. MacKenzie, Nicole A. Johnson, Nicholas D. J. Strzalkowski and Trevor A. Day*
1413 Persistence of fatigue in the absence of pathophysiological mechanisms in some patients more than 2 years after the original SARS-CoV-2 infection
*Giovanni Baldassarre, Lucrezia Zuccarelli, Thomas Favaretto, Caterina Ursella, Andrea Palomba, Paulo Cesar do Nascimento Salvador, Emanuela Sozio, Ernesto Crisafulli, Massimo Imazio, Carlo Tascini and Bruno Grassi*
1429 The effects of e-cigarette use on asthma severity in adult BALB/c mice
*Jenield J. D'abreo, Emily K. Chivers, Peter B. Noble, Luke J. Berry, Rachel R. Huxley, Arthur (Bill) W. Musk, Peter J. Franklin, Benjamin J. Mullins, Katherine R. Landwehr and Alexander N. Larcombe*
1441 The hypoxic ventilatory response and arousal burden predict the magnitude of ventilatory long-term facilitation in humans with obstructive sleep apnoea
*Jason H. Mateika, Danny Hammo, Dylan M. Kissane and Ali Azarbarzin*
1454 Peak $\dot{V}_{O_2Q}$: A new approach for the interpretation of cardiorespiratory fitness estimates
*Ben Knox-Brown, Joshua Barnes, Chris Harding, Jonathan Fuld and Karl P. Sylvester*

### VASCULAR

1466 Ageing reveals the latent effects of early life stress on respiratory and metabolic function in female rats: Novel insights into the sex-specific origins of sleep apnoea
*Danuzia Ambrozio-Marques, Loralie Mei Guay, Alicia A. Koogler, Tim D. Ostrowski and Richard Kinkead*
1483 Nitrite supplementation alleviates cerebrovascular dysfunction in chronically stressed mice, but cognitive decline remains
*Emily Burrage, Tyler Coblentz, Saina S. Prabhu, Nicole M. Eminhizer, Ryan Childers, Randall W. Bryner, Sara E. Lewis, Brooke A. Maxwell, Evan R. DeVallance, Eric E. Kelley and Paul D. Chantler*

1498 Differential contribution of T-type voltage-gated calcium channels to vascular reactivity in the aorta and renal artery of healthy rabbits
*Andrea Suarez, Sol Guerra-Ojeda, Manuel Zarzoso, Marta Serna-García, Jose M. Vila, Eva Serna and Maria D. Mauricio*

1513 The impact of healthy motion seating on lower-limb blood flow and blood pressure response to simulated long-haul air travel
*Jane Lewis, Barry J. Mcdonnell, Mark Butlin, Edward Johnston, Amira Tairi, Thomas Griffiths, Gisele Bentley, Peter Sykes and Keeron Stone*

1524 Role of S1P- and Rho-kinase signalling in age-related myogenic tone deficiency in murine resistance arteries
*Gry Freja Skovsted, Alex Aupetit, Karl Björling, Kristian Agmund Haanes, Susanne Syberg, Niklas Rye Jørgensen, Blanca I. Aldana, Hirotsugu Tsuchimochi, Mark T. Waddingham, Kristine Freude, James Todd Pearson and Lars Jørn Jensen*

### LETTER TO THE EDITOR

1543 Potential pitfalls of using a correction to normative values for the assessment of acid–base compensation during early ascent to high altitude
*Nicole V. Bushfield and Trevor A. Day*

1546 Time course of acid–base regulation at high-altitude: A century of insight
*Andrew R. Steele, Jordan D. Bird and Michael M. Tymko*

### REPLY

1550 Response to the letter from Bushfield and Day: Potential pitfalls of using a correction to normative values in the assessment of acid–base compensation during early ascent to high altitude
*Axel Kleinsasser, Benedikt Treml, Sasa Rajsic and Martin Burtscher*

1552 Response to 'Time-course of acid–base regulation at high altitude: A century of insight' by Andrew R. Steele, Jordan D. Bird and Michael M. Tymko
*Axel Kleinsasser, Martin Burtscher, Benedikt Treml and Sasa Rajsic*

Editor's Pick

Cardiac muscle sample being mechanically tested in a new high-throughput instrument. Provided by Murphy et al. Pages 632–642.
